# Male and Female Animals Respond Differently to High-Fat Diet and Regular Exercise Training in a Mouse Model of Hyperlipidemia

**DOI:** 10.3390/ijms22084198

**Published:** 2021-04-18

**Authors:** Melinda E. Tóth, Brigitta Dukay, Mária Péter, Gábor Balogh, Gergő Szűcs, Ágnes Zvara, Gábor J. Szebeni, Petra Hajdu, Márta Sárközy, László G. Puskás, Zsolt Török, Tamás Csont, László Vígh, Miklós Sántha

**Affiliations:** 1 Institute of Biochemistry, ELKH Biological Research Centre, H-6726 Szeged, Hungary; dukay.brigitta@brc.hu (B.D.); peter.maria@brc.hu (M.P.); balogh.gabor@brc.hu (G.B.); hajdu.petra@brc.hu (P.H.); torok.zsolt@brc.hu (Z.T.); vigh.laszlo@brc.hu (L.V.); santha.miklos@brc.hu (M.S.); 2 Doctoral School in Biology, University of Szeged, H-6726 Szeged, Hungary; 3 MEDICS Research Group, Department of Biochemistry, Interdisciplinary Center of Excellence, University of Szeged, H-6720 Szeged, Hungary; szucs.gergo@med.u-szeged.hu (G.S.); sarkozy.marta@med.u-szeged.hu (M.S.); csont.tamas@med.u-szeged.hu (T.C.); 4 Laboratory of Functional Genomics, ELKH Biological Research Centre, H-6726 Szeged, Hungary; zvara.agnes@brc.hu (Á.Z.); szebeni.gabor@brc.hu (G.J.S.); puskas.laszlo@brc.hu (L.G.P.)

**Keywords:** energy metabolism, hyperlipidemia, obesity, high-fat diet, exercise training, APOB-100 transgenic mice, brown adipose tissue, lipidomics

## Abstract

Inappropriate nutrition and a sedentary lifestyle can lead to obesity, one of the most common risk factors for several chronic diseases. Although regular physical exercise is an efficient approach to improve cardiometabolic health, the exact cellular processes are still not fully understood. We aimed to analyze the morphological, gene expression, and lipidomic patterns in the liver and adipose tissues in response to regular exercise. Healthy (wild type on a normal diet) and hyperlipidemic, high-fat diet-fed (HFD-fed) apolipoprotein B-100 (APOB-100)-overexpressing mice were trained by treadmill running for 7 months. The serum concentrations of triglyceride and tumor necrosis factor α (TNFα), as well as the level of lipid accumulation in the liver, were significantly higher in HFD-fed APOB-100 males compared to females. However, regular exercise almost completely abolished lipid accumulation in the liver of hyperlipidemic animals. The expression level of the thermogenesis marker, uncoupling protein-1 (*Ucp1*), was significantly higher in the subcutaneous white adipose tissue of healthy females, as well as in the brown adipose tissue of HFD-fed APOB-100 females, compared to males. Lipidomic analyses revealed that hyperlipidemia essentially remodeled the lipidome of brown adipose tissue, affecting both the membrane and storage lipid fractions, which was partially restored by exercise in both sexes. Our results revealed more severe metabolic disturbances in HFD-fed APOB-100 males compared to females. However, exercise efficiently reduced the body weight, serum triglyceride levels, expression of pro-inflammatory factors, and hepatic lipid accumulation in our model.

## 1. Introduction

Obesity is one of the most common risk factors for the leading causes of death in modern societies, highlighting the need for its prevention and therapy. Obesity and overweight cause complex health problems affecting the whole body. Inappropriate nutrition and a sedentary lifestyle increase the likelihood of developing obesity and related diseases, such as cardiovascular disorders and metabolic syndrome. Metabolic syndrome is a cluster of symptoms that are the most common risk factors for cardiac diseases and type 2 diabetes. According to the International Diabetes Federation [1], the diagnostic criteria for metabolic syndrome are abdominal obesity and at least two of the following factors: increased serum triglyceride level, decreased high-density lipoprotein (HDL) cholesterol, elevated blood pressure, and raised fasting serum glucose. The coexistence of these alterations leads to a higher prevalence of cardiovascular dysfunctions (two- to three-fold increase in risk), type 2 diabetes (3.5–5-fold increase), and mortality [2,3]. The incidence of these different components and their combinations depends on several factors, including non-modifiable aspects like genetic background and age, while others, such as diet and physical inactivity, can be modified by the patient [3,4].

Maintaining normal energy homeostasis and healthy metabolism requires a strictly regulated cooperation between different organs, such as the liver and adipose tissues. Mice express two major types of adipocytes: white and brown, and based on their location, subcutaneous and visceral adipose tissues can be distinguished [5]. White adipocytes have a single large lipid droplet in the cytoplasm, and are specialized for energy storage in the form of triglycerides [5,6]. However, adipose tissues are not only the major energy reservoirs, but they also have important endocrine functions, like the production of various hormone-like molecules and cytokines, called adipokines. Thereby, the adipose tissues are involved in the regulation of whole-body metabolism and energy homeostasis [2,6,7]. Brown adipocytes contain many smaller lipid droplets and numerous mitochondria, having an important role in non-shivering thermogenesis [6]. The thermogenic activity of brown adipose tissue (BAT) is especially important in small mammals and newborn humans, and helps to maintain body temperature in a cold environment. Previously, it was thought that BAT is absent, or at least it has no relevance, in adults. However, it was shown that adults also have metabolically active BAT, and it may play an important role in energy homeostasis [8]. Moreover, exercise-induced “browning” of white adipose tissues (WATs) might also contribute to whole-body energy utilization that prevents fat storage, therefore, it could be a potential target to control obesity and related metabolic abnormalities [6,9]. The metabolic and thermogenic activities of the different types of adipocytes are influenced, among many other factors, by the so-called hepatokines, secreted by liver cells. In turn, a variety of endocrine and cytokine molecules derived from adipose tissues are also involved in the regulation of liver metabolism. Therefore, a significant increase in WAT, especially in the abdominal cavity, plays an important role in the development of liver abnormalities related to metabolic syndrome, such as non-alcoholic fatty liver disease [10]. According to the portal theory, free fatty acids and pro-inflammatory cytokines produced by visceral WAT, such as interleukin- 1β (IL1β), directly influence liver metabolism, and therefore these may be involved in the development of hepatic insulin resistance and steatosis [10,11,12].

The simplest strategies to prevent and treat metabolic syndrome include a healthy diet and regular physical activity. It has been demonstrated that low levels of aerobic capacity are associated with an increased risk of metabolic syndrome and cardiovascular diseases. On the other hand, increasing evidence supports that exercise training is able to decrease blood pressure and insulin resistance, and positively influences the serum lipid profile [13,14]. Similar to obesity, exercise has a whole-body effect, and while the benefits of regular training are generally accepted, the exact cellular processes explaining these benefits have not been fully elucidated.

Therefore, our study aimed to analyze the morphological and gene expression patterns of the liver and different types of adipose tissues in response to regular exercise in healthy (wild type on a normal chow diet) and hyperlipidemic, high-fat diet (HFD)-fed APOB-100-overexpressing mice. Apolipoprotein B-100 (APOB-100) is the major protein component of low-density lipoproteins (LDLs) and very low-density lipoproteins (VLDLs). Overexpression of APOB-100 leads to an increased LDL/HDL ratio, and the development of a serum lipid profile resembling that of humans. Therefore, these transgenic mice are more susceptible to diet-induced hyperlipidemia and related cardiovascular dysfunctions [15,16]. However, while these alterations are well accepted and APOB-100 mice serve as a validated model of human hyperlipidemia and atherosclerosis, much less is known about the functional and morphological alterations induced by an HFD in other tissues in these transgenic mice. It has been shown that the overproduction of the human APOB-100 protein in the liver leads to endoplasmic reticulum (ER) stress and insulin resistance and induces hepatic steatosis [17]. Our current study focused on obesity-related dysregulation of lipid and glucose metabolism, as well as on the morphology and gene expression profiles of the liver and different adipose tissues. Using the HFD-fed APOB-100 animal model, we examined whether moderate-level regular exercise training could ameliorate these related alterations. Male and female animals were studied in separate groups because of the remarkable sex differences previously reported for human patients, regarding, for example, the susceptibility to metabolic syndrome or cardiovascular diseases [3,4]. Our results confirm that male APOB-100 transgenic animals show more severe metabolic disturbances after 7 months of an HFD compared to females, with regard to, for example, fasting glucose, serum triglyceride (TG), tumor necrosis factor α (TNFα) levels, and hepatic steatosis. However, daily exercise training efficiently reduced the rate of the increase in body weight, serum triglyceride level, and the expression of pro-inflammatory factors, and also prevented lipid accumulation in the liver in our model.

## 2. Results

### 2.1. Sex Differences in the Prevalence of Metabolic Disturbances in HFD-Fed APOB-100 Transgenic Mice

#### 2.1.1. Body Weight, Serum Glucose, TG, Leptin, and TNFα Levels

**Figure 1:**
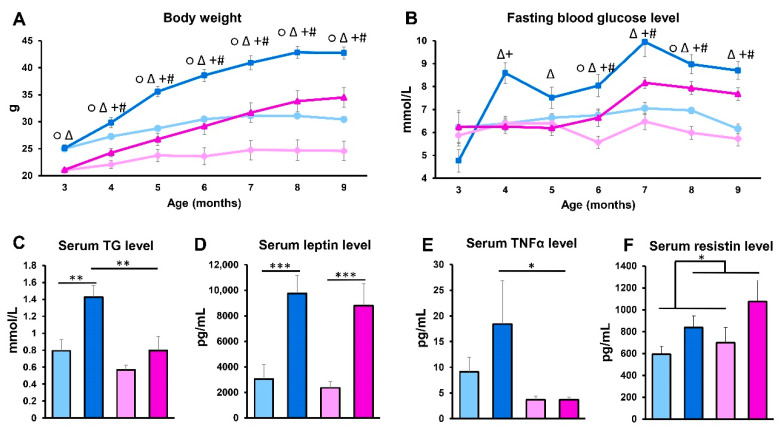
Body weight and serum parameters. (**A**) Body weight and (**B**) serum glucose levels were measured at baseline and monthly thereafter during the experiment (*n* = 10–12 animals/group). At the end of the experiment, serum samples were collected. (**C**) Serum TG levels were measured using an enzymatic colorimetric assay kit (*n* = 10–12 animals/group). (**D**) Serum concentrations of leptin, (**E**) TNFα, and (**F**) resistin were quantified using a MILLIPLEX^®^ Mouse Adipokine Multiplex Immunoassay (*n* = 10 animals/group). Values are presented as mean ± SEM; ○ indicates wild-type male group vs. wild-type female group; Δ indicates APOB-100 male group vs. APOB-100 female group; + indicates APOB-100 male group vs. wild type male group; # indicates APOB-100 female group vs. wild type female group; * denotes *p* < 0.05; ** denotes *p* < 0.01; *** denotes *p* < 0.001. WT/ND, wild-type mice on normal diet; APOB/HFD, APOB-100 transgenic mice on high-fat diet; TG, triglyceride.

For our experiments, we used HFD-fed APOB-100 transgenic mice (APOB/HFD), a model of hyperlipidemia, and wild-type C57B6 mice fed with normal chow as a healthy control (WT/ND). The HFD of the transgenic animals was started at the age of 3 months (Figure S1B). Male and female animals were studied in separate groups (Figure S1A). Body weight and fasting glucose levels were measured at baseline and monthly thereafter throughout the experiment. The body weight of APOB-100 transgenic mice on the HFD constantly increased in a more pronounced manner compared to the controls, both in male and female groups, although the females had a lower body weight at each time point (Figure 1A). Fasting blood glucose levels of HFD-fed APOB-100 animals were also elevated throughout the experiment compared to controls. In the APOB-100 male group, serum glucose showed a significant increase compared to wild-type controls as early as one month after starting the dietary intervention, while in transgenic females, fasting glucose started to become elevated after 3 months of the high-fat diet (Figure 1B). After 7 months of the HFD, serum TG levels were also compared, showing a two-fold higher level of TG in HFD-fed APOB-100 males compared to wild-type, normal diet controls and APOB-100 females (*p* < 0.01). Interestingly, in females, no significant difference between the HFD-fed APOB-100 group and wild-type, normal diet controls could be detected in serum TG concentrations (Figure 1C). Serum leptin levels at 7 months were more than three-fold higher in HFD APOB-100 transgenic animals, both in males and females, compared to the wild-type, normal diet control groups (*p* < 0.001) (Figure 1D). Serum TNFα concentrations, in general, were higher in males than in females, showing a further increase in hyperlipidemic males, while in females, no differences between the APOB-100 HFD and wild-type, normal diet groups were detected (Figure 1E). Moreover, serum resistin concentrations were higher in hyperlipidemic groups than in healthy animals (Figure 1F), while we could not detect significant differences between the groups regarding serum insulin levels (Figure S2).

#### 2.1.2. Liver and Brown Adipose Tissue Morphology

**Figure 2:**
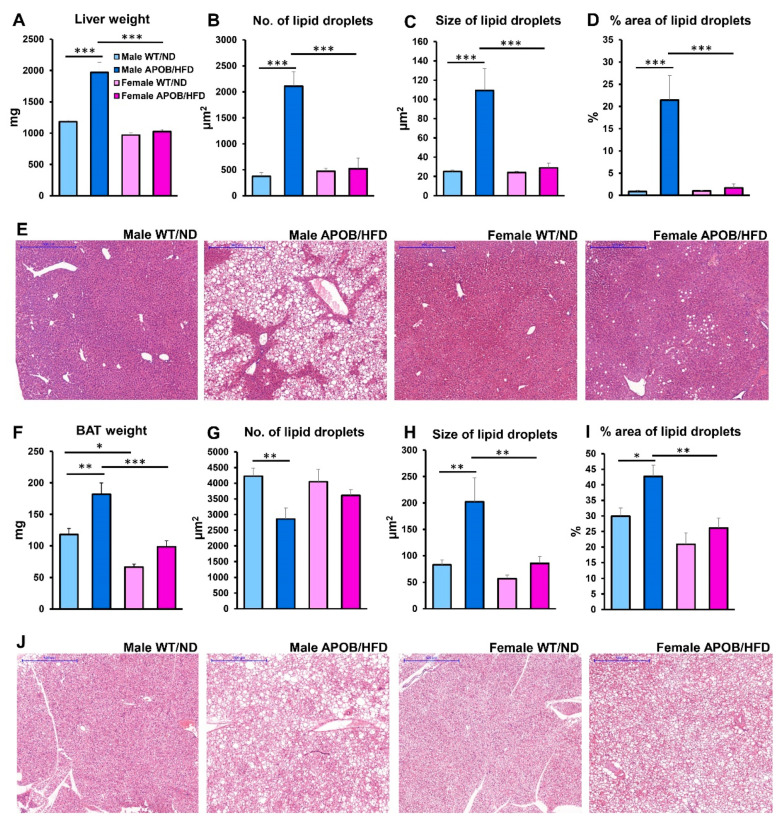
Lipid accumulation in the liver and brown adipose tissue. (**A**) Weight of the liver and (**F**) BAT were measured. Then, after formalin fixation, tissues were embedded in paraffin. (**E**) Liver and (**J**) BAT sections were stained with hematoxylin and eosin. Scale bar: 500 μm. The number (**B**—liver, **G**—BAT), size (**C**—liver, **H**—BAT), and area (**D**—liver, **I**—BAT) of lipid droplets were quantified using ImageJ software (*n* = 6 animals/group). Values are presented as mean ± SEM; * denotes *p* < 0.05; ** denotes *p* < 0.01; *** denotes *p* < 0.001. WT/ND, wild-type mice on normal diet; APOB/HFD, APOB-100 transgenic mice on high-fat diet; BAT, brown adipose tissue.

At the end of the experiment, the liver and adipose tissues were isolated for further investigations. The liver of male APOB-100/HFD animals was found to be 66% heavier compared to the wild-type, normal diet controls. Interestingly, the liver of female animals was equal, irrespective of genotype and differing diet (Figure 2A). The weight of the interscapular brown adipose tissue was higher in male HFD-fed transgenic animals than in the corresponding wild-type, normal diet control group (Figure 2F). In accordance with body weight changes, hematoxylin–eosin (HE) staining of the tissue sections revealed large vacuoles in the liver (Figure 2E) and in the brown adipose tissue (Figure 2J) of transgenic male animals, suggesting triglyceride accumulation in response to the high-fat diet. The number and size of lipid droplets were determined by ImageJ software. In the healthy liver of WT/ND animals, as well as in the liver of HFD-fed APOB-100 females, a minimal amount of lipid droplets was detected. However, the number and size of lipid droplets were dramatically elevated in the liver of HFD-fed APOB-100 males, associated with an overall increase in the area of lipid accumulation compared to wild-type, normal diet controls, as well as compared to the HFD-fed APOB-100 female group (*p* < 0.01). In contrast, the number and the size of lipid droplets only slightly differed between the two female groups (Figure 2B–D). Regarding brown adipose tissue, it should be noted that brown adipocytes normally contain a large number of small lipid droplets in their cytoplasm. In the BAT of hyperlipidemic male animals, the lipid droplets were found to be larger, but their number was lower compared to the healthy control groups, as well as compared to the HFD-fed APOB-100 female group. Regarding both the number and size of lipid droplets, the difference between the male groups (HFD-fed APOB-100 males vs. wild-type, normal diet-fed controls) reached statistical significance (*p* < 0.01). With respect to the size of lipid droplets, the difference between HFD-fed APOB-100 males and females was also significant (*p* < 0.01). Regarding female animals only, the difference between HFD-fed APOB-100 and wild-type, normal diet-fed mice was not pronounced (Figure 2G–I).

#### 2.1.3. Differences in Gene Expression Patterns

**Figure 3:**
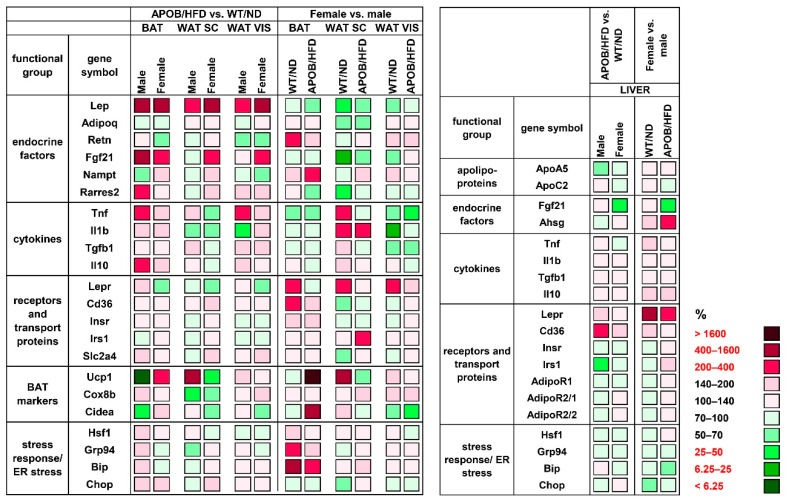
Heatmap of relative gene expression differences in adipose tissues and in the liver in response to hyperlipidemia. The relative expressions of different endocrine and cytokine factors, receptors, and transport proteins were studied in adipose tissues and in the liver of healthy and hyperlipidemic mice using qPCR (*n* = 6 animals/group). For APOB/HFD vs. WT/ND comparisons, the relative expression of target genes in transgenic animals was compared to the expression levels detected in wild-type animals (results are given as a percentage, where WT/ND groups = 100%). For the female vs. male comparison, the relative expression of target genes in females was compared to the expression levels detected in males (results are given in percentages, where the male groups = 100%). WT/ND, wild-type mice on normal diet; APOB/HFD, APOB-100 transgenic mice on high-fat diet; BAT, brown adipose tissue; WAT SC, subcutaneous white adipose tissue; WAT VIS, visceral white adipose tissue.

To analyze the effect of hyperlipidemia on the expression of genes involved in the regulation of glucose and lipid metabolism, thermogenesis, and inflammation, we isolated total RNA from the liver and from the different types of adipose tissues (BAT, subcutaneous WAT – WAT SC, visceral WAT – WAT VIS), and gene expression levels were measured by qPCR. The differences between HFD-fed APOB-100 animals and wild-type, normal diet-fed controls, as well as sex differences, were compared. First, we analyzed the effect of hyperlipidemia by comparing the values for the APOB-100 groups to the wild-type, normal diet-fed controls (APOB/HFD vs. WT/ND; for all comparisons WT/ND = 100%). The gene encoding leptin (*Lep*) showed a two to six times higher expression level both in transgenic male and female animals compared to the controls in all the adipose tissues studied (male BAT: 657%, *p* < 0.01; female BAT: 420%, *p* < 0.05; male WAT SC: 221%, *p* < 0.01; female WAT SC: 479%, *p* < 0.05; male WAT VIS: 318%, *p* < 0.01; female WAT VIS: 474%, *p* < 0.01; Figure 3 and Table S1). The expression level of *Fgf21* (fibroblast growth factor 21) was also significantly increased in the BAT of hyperlipidemic groups, both in males and females (403%, *p* < 0.05 and 365%, *p* < 0.01, respectively), while in visceral WAT, it was found to be increased in females only (234%, *p* < 0.05). Moreover, we found a two-fold increase in the expression level of the gene encoding adipokine chemerin (*Rarres2*- retinoic acid receptor responder 2) in the BAT of APOB/HFD males compared to WT/ND males (*p* < 0.05). Among the examined cytokines, *Tnf* showed a higher gene expression level in the BAT (203%, *p* < 0.05) and visceral WAT (218%, *p* < 0.05) of male HFD-fed transgenic animals, while only smaller, non-significant differences were detected for *Tnf* expression in the adipose tissues of female animals. The *Ucp1* gene, encoding uncoupling protein-1, the most important marker of thermogenesis, showed an amazingly low expression level in the BAT of transgenic male animals compared to WT males (5%, *p* < 0.05). In contrast, *Ucp1* expression was significantly higher in the BAT of APOB/HFD females than in healthy controls (239%, *p* < 0.01). More interestingly, contrary differences were found for the subcutaneous white adipose tissue samples: *Ucp1* expression was increased in APOB/HFD males and decreased in the APOB/HFD female group compared to the controls, although these differences were not statistically significant (570%, *p =* 0.0762, and 48%, *p =* 0.479, respectively). In the liver, the expression level of *Cd36* (cluster of differentiation 36) was significantly increased (240%, *p* < 0.01), while *Irs1* (insulin receptor substrate 1) expression was decreased (49%, *p* < 0.001) in male APOB/HFD mice compared to healthy controls (Figure 3, Table S2).

We also aimed to reveal potential sex differences, thus, we performed a direct comparison of female vs. male groups (Figure 3, Tables S1 and S2). For this relation, the values for the corresponding male animals (APOB/HFD or WT/ND) were considered as 100%. *Lep* expression in the subcutaneous WAT was found to be lower in female animals compared to males (WT/ND: 26%, *p* = 0.069; APO/HFD: 56%, *p* < 0.05). Lower mRNA levels of *Fgf21* (20%, *p* < 0.05) and *Rarres2* (47%, *p* < 0.05) were detected in the subcutaneous WAT of WT females compared to WT males. On the other hand, the expression of *Retn*, encoding resistin (226%, *p* < 0.05), and *Nampt*, (nicotinamide phosphoribosyltransferase) encoding visfatin (367%, *p* < 0.01), was higher in the BAT of WT and APOB-100 females, respectively, when compared to the corresponding male groups. Pro-inflammatory cytokine expression tended to be lower in the visceral WAT of female animals: mRNA for *Tnf* showed a significantly lower level in transgenic females (42%, *p* < 0.05), while mRNA for *Il1b* was decreased in WT females (24%, *p* < 0.01) compared to the corresponding male groups. Regarding the markers of thermogenesis, the level of *Ucp1* expression was found to be 30 times higher in the BAT of APOB/HFD females (3200%, *p* < 0.01), and was also significantly elevated in the subcutaneous WAT of WT females (644%, *p* < 0.05) compared to the corresponding male groups. The mRNA expression of *Lepr* (encoding the leptin receptor) was consistently higher in all the examined tissues of healthy female animals. The difference was significant in the BAT (247%, *p* < 0.05) and visceral WAT (255%, *p* < 0.01) of WT female mice compared to WT males. Moreover, *Lepr* mRNA expression was dramatically increased in female liver samples compared to both the healthy and hyperlipidemic male groups (521%, *p* < 0.01; 281%, *p* < 0.05). Interestingly, the expression level of the *Ahsg* (alpha 2-HS glycoprotein) gene, encoding fetuin, was twice as high in the liver samples of APOB-100 females than in those of APOB-100 males (*p* < 0.05).

#### 2.1.4. Differences in BAT Lipidomic Patterns

A very strong correlation was revealed between body weight and BAT mass (*r* = 0.816, *p* = 1.5 × 10^−11^; Figure S3) and the most remarkable differences in gene expression patterns occurred in BAT (Figure 3). Therefore, we hypothesized that the lipid metabolism of BAT was also affected by hyperlipidemia, and it was supposed to differ in sexes. To test this hypothesis, we performed mass spectrometry-based shotgun lipidomic analyses on BAT lipid extracts. Our lipidomic platform allowed the broad-range coverage of the BAT lipidome; we identified and quantified ca. 275 lipid molecular species encompassing 21 lipid classes, including both membrane and storage lipids (Table S3).

**Figure 4:**
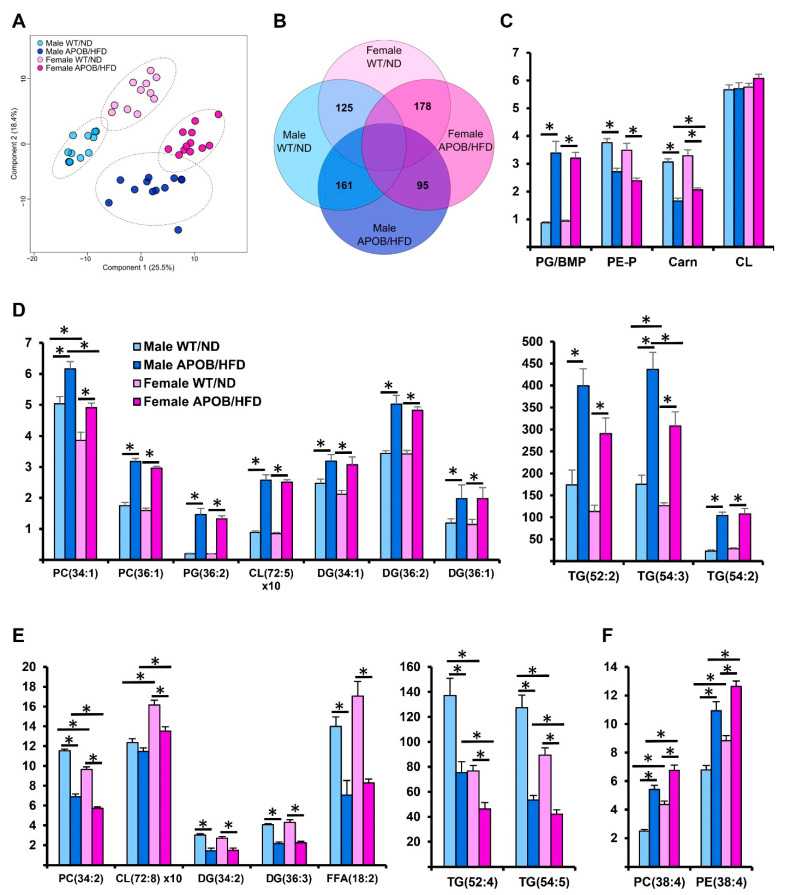
Differences in lipidomic patterns in response to hyperlipidemia and sex differences in the sedentary groups. (**A**) Partial least squares discriminant analysis (PLS-DA) score plot of lipidomic datasets based on the whole lipidome. Dashed circles display 95% confidence regions. (**B**) Venn diagram displaying the number of statistically different components. (**C**) Differences in lipid classes, (**D**) increase in oleic acid-containing lipid species, (**E**) decrease in linoleic acid-containing lipid species, and (**F**) increase in arachidonic acid-containing components due to hyperlipidemia (*n* = 10–12 mice per group). * denotes *p* < 0.05. PC, diacyl phosphatidylcholine; PE, diacyl phosphatidylethanolamine; PE-P, alkenyl-acyl phosphatidylethanolamine; PG, phosphatidylglycerol; BMP, bis(monoacylglycero)phosphate; CL, cardiolipin; Carn, carnitine; DG, diacylglycerol; TG, triacylglycerol; FFA, free fatty acid.

Lipidomic data expressed as mol% of membrane lipids were subjected to partial least squares discriminant analysis (PLS-DA), which revealed very clear separations between the wild-type and HFD-fed transgenic animals (Figure 4A). Importantly, the separations remained practically unchanged upon considering membrane lipids only, which accounted for no more than 8% of the BAT lipidome (Figure S4). Next, we compared the molecular species patterns for the different groups. We prepared a Venn diagram to visualize the number of statistically significant between-group differences (Figure 4B). In response to hyperlipidemia, 71% of the quantified lipid species changed significantly in either the male or female group. APOB-100 animals displayed 161 and 178 different species compared to WT males and females, respectively. On the other hand, the hyperlipidemic response displayed similar patterns in both sexes, and indeed, 73% of the significantly altered components were common features (Figure S5).

At the lipid class level, the most remarkable difference was the prominent increase in the relative amounts of phosphatidylglycerol (PG) and/or bis(monoacylglycero)phosphate (BMP), while plasmalogen phosphatidylethanolamine (PE-P) and carnitine (Carn) were substantially decreased in both sexes (Figure 4C). Interestingly, the level of cardiolipin (CL) was similar in all groups (Figure 4C). Regarding lipid species, hyperlipidemia completely reshaped the molecular species profiles of practically all individual lipid classes (Table S3). The response could be characterized by the relative increase in oleic (18:1)-containing (Figure 4D) and decrease in linoleic acid (18:2)-containing components (Figure 4E) (for fatty acyl-resolved structures, see Table S4). The large increase in 18:1-containing TG species supported the appearance of larger, vacuole-like lipid droplets in the BAT of APOB-100 HFD-fed animals well (Figure 2J). The levels of such species correlated strongly with BAT mass (e.g., *p* = 0.83 for TG(54:3) and *p* = 0.82 for TG(52:2)). In addition, we detected a considerable accumulation of the arachidonic acid (20:4)-containing species in the membrane lipid pool (Figure 4F). As a result of these concomitant changes, the double bond index (DBI), a measure of unsaturation, did not change in the comparison of WT and hyperlipidemic animals (Table S3).

In addition to the effects of hyperlipidemia, the samples grouped into easily distinguishable clusters based on sex differences (Figure 4A). The number of lipid constituents distinguishing male and female mice was around 100 in both the WT and APOB-100 animals (Figure 4B). A major sex-specific feature was the significantly higher ratio of membrane to storage lipids in females compared to males (Table S3). Regarding lipid species, female BAT was revealed to possess a larger amount of arachidonic acid, especially in combination with stearic acid (Figure 4F); this resulted in a slightly but significantly higher membrane unsaturation in the female groups (Table S3). It is also noteworthy that the level of the major CL species, the homosymmetric tetralinoleoyl CL(72:8), was significantly higher in the female BAT than in BAT of males (Figure 4E). Furthermore, the levels of lysolipids, lysophosphatidylcholine (LPC) and lysocardiolipin(LCL), were also higher in the female groups.

### 2.2. Regular Exercise Training Efficiently Ameliorated Certain Features of Metabolic Syndrome

#### 2.2.1. Analysis of Body Weight, Serum Biochemical Parameters, and the Morphology of Liver and Brown Adipose Tissue

**Figure 5:**
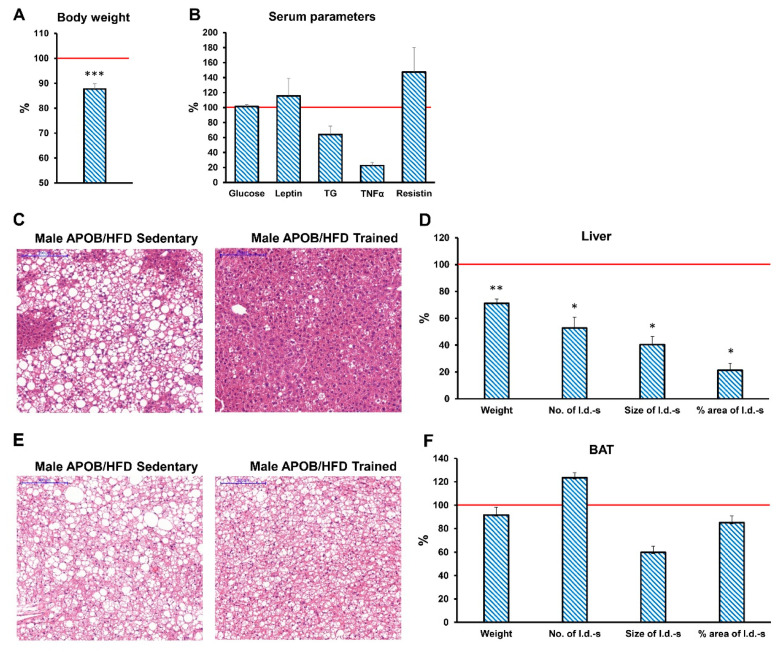
Effects of exercise training in HDF-fed male APOB-100 transgenic mice. At the end of the experiment, (**A**) body weight was measured and serum samples were collected to determine the concentration of (**B**) serum glucose, leptin, TG, TNFα, and resistin. Lipid accumulation in the liver and brown adipose tissue was also analysed. (**C**) Liver and (**E**) BAT sections were stained with hematoxylin and eosin. Scale bar: 200 μm. Weight of the (**D**) liver and (**F**) BAT were measured and the number, size, and area of lipid droplets in the (**D**) liver and in the (**F**) BAT were quantified using ImageJ software. Blue striped columns represent the differences in the given value in regularly trained APOB-100/HFD male mice, expressed as the percentage of the corresponding value for sedentary APOB-100/HFD males. *n* = 12 mice per group for body, liver, and BAT weight and serum glucose measurements, *n* = 10 mice per group for serum leptin, TNFα, and resistin concentration measurements, *n* = 6 mice per group for the quantification of lipid accumulation. Values are presented as mean ± SEM; * denotes *p* < 0.05; ** denotes *p* < 0.01; *** denotes *p* < 0.001. WT/ND, wild-type mice on normal diet; APOB/HFD, APOB-100 transgenic mice on high-fat diet; BAT, brown adipose tissue; l.d., lipid droplet.

In order to study the effect of regular exercise training on hyperlipidemia-induced alterations in our model, the animals were randomly divided into sedentary control and exercise groups, and the latter group was subjected to daily treadmill running, five times a week for 7 months. As expected, the body weight of transgenic, HFD-fed animals was significantly lower in the regularly trained group, both for males and females, compared to sedentary controls (Figure 5A and Figure S6A). In contrast, serum glucose and leptin levels were not influenced by daily exercise training in the HFD groups (Figure 5B and Figure S6B). In male HFD-fed APOB-100 animals, serum TG and TNFα levels were lower, while the resistin concentration was higher in the trained group than in sedentary controls, however, the difference was not statistically significant (Figure 5B). While the weight of interscapular brown adipose tissue was unaffected by regular exercise (Figure 5E,F), the increase in liver weight was less pronounced in male APOB/HFD animals subjected to regular exercise training (Figure 5D). In parallel, lipid accumulation and the number, size, and total area of lipid droplets were also lower in the liver of male APOB/HFD animals with regular exercise training compared to sedentary controls, as indicated by HE staining (Figure 5C) and quantification of the results (Figure 5D).

#### 2.2.2. Differences in Gene Expression Patterns

**Figure 6:**
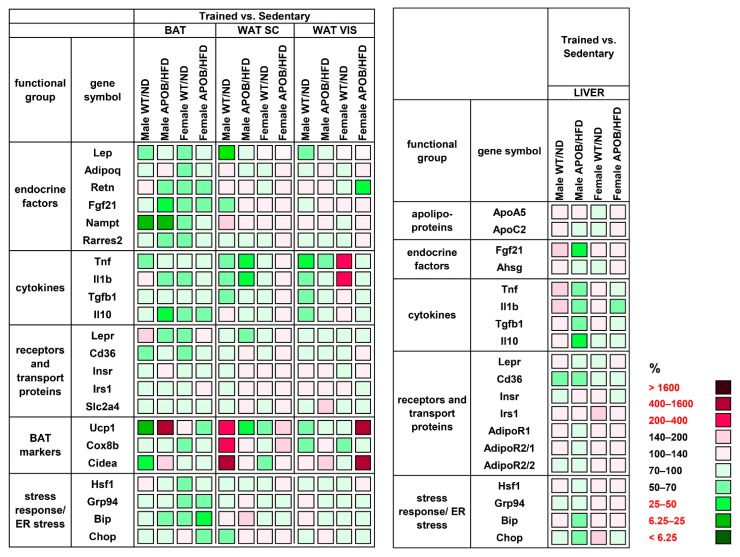
Heatmap of relative gene expression differences in adipose tissues and in the liver in response to exercise training. Relative expressions of different endocrine and cytokine factors, receptors, and transport proteins were studied in the adipose tissues and in the liver of healthy and hyperlipidemic mice using qPCR (*n* = 6 animals/group). The relative expression of target genes in sedentary animals was compared with the expression levels detected in the corresponding trained groups (results are given as a percentage, where the corresponding value for the sedentary groups = 100%). WT/ND: wild-type mice on normal diet; APOB/HFD, APOB-100 transgenic mice on high-fat diet; BAT, brown adipose tissue; WAT SC, subcutaneous white adipose tissue; WAT VIS, visceral white adipose tissue.

Our gene expression analyses revealed a significantly lower *Lep* mRNA level in the subcutaneous (39%, *p* < 0.05) and visceral (51%, *p* < 0.05) WAT of trained WT male animals compared to the sedentary group (Figure 6, Table S5). Expression of the examined cytokines mostly showed decreasing values in the trained groups (Figure 6, Tables S5 and S6). Among them, the mRNA levels of *Tnf* and *Il1b* in the subcutaneous WAT of trained APOB/HFD males were half of those measured in the corresponding sedentary group (*p* < 0.05), while trained WT males showed reduced *Tnf* levels in the visceral WAT (48%, *p* < 0.05) compared to their corresponding sedentary counterparts. A surprising exception is the significantly increased level of *Tnf* in the visceral WAT of WT female mice (230%, *p* < 0.05). Moreover, we detected a significant decrease in the expression of BAT markers, *Ucp1* and *Cidea*, (cell death-inducing DFFA-like effector A) in the BAT of trained WT males (18%, *p* < 0.05; 31%, *p* < 0.05, respectively). Interestingly, the same markers were rather increased in the subcutaneous WAT of the same group, where *Cidea* showed a more than six-fold increase in trained animals compared to sedentary ones (*p* < 0.05). On the other hand, contrary differences indicating changes in the opposite direction were observed in the case of APOB-100 males, although these differences were not statistically significant (Figure 6, Table S5).

#### 2.2.3. Differences in BAT Lipidomic Patterns

**Figure 7:**
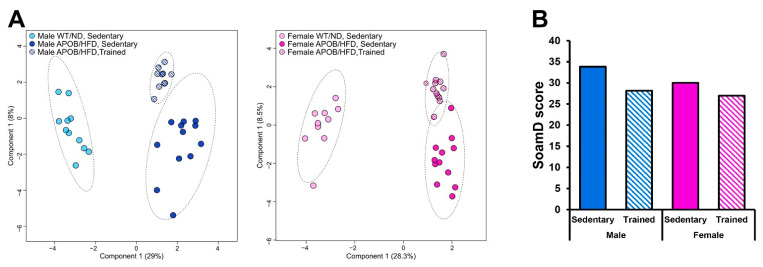
Lipidomic changes in response to exercise. (**A**) sPLS-DA score plots of lipidomic datasets; left panel—males, right panel—females. Dashed circles display 95% confidence regions. (**B**) SoamD scores (sum of absolute mol% difference for membrane lipids) for the APOB-100 transgenic groups compared to the corresponding sedentary WT groups.

To account for the effect of exercise, we compared the BAT lipidomes of sedentary WT, sedentary APOB/HFD, and regularly trained APOB/HFD animals. sPLS-DA score plots showed clear separations for these groups in both sexes (Figure 7A). We also calculated the sum of the absolute mol% difference (SoamD score) [18] for membrane lipids relative to the sedentary WT groups (Figure 7B). Using this approach, we recorded the largest lipidome deviation for the male sedentary APOB/HFD group, where the SoamD value accounted for 34% of the membrane lipidome. Regular exercise reduced the distance between the trained APOB/HFD and sedentary WT/ND animals as compared to the sedentary APOB/HFD mice in both sexes; its effect was more pronounced in males. Next, we searched for lipid molecular species which differed significantly and simultaneously between sedentary WT/ND and APOB/HFD as well as between sedentary and regularly trained APOB/HFD groups in either male or female mice. We found 80 molecular species that fulfilled this requirement (Table S3). Many selected molecular species were membrane lipids, which, in several cases, displayed contrary differences indicating opposite changes for the two comparisons. Nineteen percent of the selected set was common to males and females, such as LPC(20:4), phosphatidylcholine (PC) (38:4), or PG(38:5), but the majority was sex specific. For example, numerous linoleic acid-containing components, such as PC(34:3), PC(34:2), PC(36:3), or PE(34:2), differed only in males, while certain lysolipid, PG, and diacylglycerol (DG) species differed only in females.

## 3. Discussion

APOB-100-overexpressing transgenic mice show hyperlipidemia [15,19,20] and numerous studies support that an elevated serum cholesterol level induced by a high-fat/high-cholesterol diet leads to cardiovascular dysfunction in these animals. Therefore, the high-fat/high-cholesterol diet-fed APOB-100 mouse strain is a widely accepted and validated model of human hyperlipidemia and atherosclerosis [15,16,21]. In this study, we used this transgenic animal model to explore hyperlipidemia-related alterations other than cardiovascular manifestations, such as obesity, increased serum glucose, or lipid accumulation in the liver. As expected, a high-fat diet significantly increased the body weight of APOB-100 animals. Although the females had a lower body weight at each time point compared to males in the APOB-100 groups, the rate of weight gain was significant for both sexes. The increase in body weight was accompanied by the accumulation of subcutaneous and visceral adipose tissues. White adipose tissues function not only as energy reservoirs, but also as endocrine and immune organs [2,7]. Moreover, brown adipose tissue is able to consume a significant amount of calories via non-shivering thermogenesis, further influencing whole-body metabolism and energy homeostasis.

Therefore, we analyzed the expression of inflammatory markers and other regulators of metabolism in the different types of adipose tissue, including interscapular brown adipose tissue and subcutaneous and visceral white adipose tissues. Our qPCR results indicate increased *Lep* expression in the adipose tissues of APOB/HFD animals in both sexes, however, it was found to be lower in the subcutaneous WAT of females upon a direct comparison of the sexes. Serum leptin levels were also significantly increased in the hyperlipidemic transgenic group, which is in accordance with previous studies showing that serum leptin concentrations correlate with fat mass [22,23]. Leptin is one of the most abundant adipokines, secreted mainly by the adipose tissues, and plays a central role in the regulation of energy homeostasis and body weight by alleviating appetite through the hypothalamic leptin receptors [24,25]. Moreover, leptin receptors are expressed by the liver and the adipose tissues as well [22,23]. Leptin has been shown to suppress insulin sensitivity of adipocytes and inhibit lipid synthesis and accumulation [23]. We found a higher level of leptin receptor mRNA in the adipose tissues of healthy (WT/ND) female mice compared to males, which may partly explain sex-specific differences in susceptibility to certain metabolic abnormalities. The expression level of the gene encoding fibroblast growth factor 21 (FGF21), an important regulator of energy metabolism and insulin sensitivity, was elevated in BAT and visceral WAT of transgenic females. In contrast, in males, *Fgf21* expression was significantly increased in BAT only. FGF21 expression was found to be induced in white and brown adipocytes in response to cold exposure, suggesting that FGF21 might promote the beiging of white adipose tissue and might increase the activity of brown adipocytes [26]. Our results indicate that hyperlipidemia is also accompanied by increased *Fgf21* expression in adipose tissues, especially in female mice. However, it does not correlate with the expression of thermoregulatory markers, suggesting that other factors are also involved in the regulation of thermogenic activity of adipose tissues.

Overall, adipocyte hypertrophy disturbs the level and the function of secreted adipokines, and facilitates the production of pro-inflammatory molecules, finally leading to low-grade systemic chronic inflammation [5,7,27]. Indeed, we measured increased serum TNFα concentration in response to hyperlipidemia, however, only in the male group. Moreover, adipokines are also involved in the regulation of insulin signaling, and the imbalance of these factors may have an important role in the development of type 2 diabetes [2,28]. Although we detected increased fasting blood glucose in the HFD-fed APOB-100 groups in both sexes, in this regard, a delay was detected in females, who showed elevated glucose levels after 3 months of the diet only, suggesting that females are less susceptible to insulin resistance in the first few months of a high-fat diet. These results are in accordance with previous findings demonstrating that estrogen can reduce oxidative stress and systemic inflammation and improve insulin resistance [29,30,31]. Insulin resistance is frequently associated with hypertriglyceridemia [2,32]. Accordingly, serum triglyceride levels were elevated in our hyperlipidemic transgenic model compared to normal diet-fed, wild-type animals, especially in males.

The liver also has a crucial role in lipid metabolism as it participates in the accumulation and distribution of lipids and lipoproteins. Obesity, diabetes, and metabolic syndrome are important risk factors for non-alcoholic fatty liver disease, which is characterized by an excess triglyceride accumulation in hepatic tissues, leading to inflammation and fibrosis [33]. In our experiment, increased serum lipid levels triggered the accumulation of fatty acids and triglycerides in the liver of APOB-100 transgenic animals, as reflected by liver weight changes: in sedentary transgenic males, the weight of the liver was significantly higher than in the wild-type groups. Moreover, histological analyses revealed signs of hepatic steatosis in the liver of HFD-fed APOB-100 animals. However, unlike in males, neither liver weight nor lipid accumulation was significantly increased in APOB-100 females. Among many predisposing factors, hepatic leptin resistance is reported to have a major role in hepatic lipid accumulation [34], as disruption of leptin receptor signaling in the liver in vivo increases hepatic lipid accumulation [35], while upregulating leptin receptor expression was efficient in reducing hepatic steatosis and triglyceride content [36]. In accordance with these previous findings, we detected a significantly higher level of *Lepr* expression in the liver of female animals compared to males, both in the wild-type and in the transgenic groups. Thus, we can suppose that higher hepatic leptin sensitivity might contribute to the lower level of steatosis and serum triglyceride level detected in HFD-fed females as opposed to males. Furthermore, earlier results showed that estrogen can increase insulin sensitivity and triglyceride export in the liver, resulting in reduced hepatic lipid accumulation [37].

Another phenomenon might also predispose APOB-100 male mice to certain metabolic abnormalities induced by an HFD. As mentioned above, the main function of BAT is generating heat (instead of ATP) from energy, via uncoupled respiration. This process of non-shivering thermogenesis is mediated by a major thermogenic factor called uncoupling protein-1 (UCP1) [6]. In addition, in response to certain conditions, such as cold exposure or exercise training, “browning” of WAT is also observed. The resultant beige fat is characterized by an increased expression of the most important thermoregulatory markers UCP1, cell death-inducing DFFA-like effector A (CIDEA), and cytochrome C oxidase subunit VIIIb (COX8b) [38]. Surprisingly, we found that *Ucp1* expression in the subcutaneous WAT of healthy (WT/ND) female mice is six times higher than that in males, likely because of the higher thermal preference of females compared to males. This may be associated with sex differences in body weight [39], and is probably compensated by more intensive thermogenesis in females. Accordingly, females might need to burn more calories than males in order to maintain their body temperature under the same circumstances. This may contribute to a slower development of HFD-induced metabolic disturbances in female animals, as suggested by, for example, the delayed increase in fasting glucose. Furthermore, we detected sex-specific alterations in the morphology and gene expression profile of BAT in response to hyperlipidemia. HE staining revealed significant lipid accumulation in the BAT of transgenic animals. As a result, BAT structure appeared more similar to WAT, with adipocytes possessing fewer but larger lipid droplets. Although these differences were observed in both sexes in the APOB-100 group compared to normal diet-fed, wild-type controls, the differences were much more pronounced in males than in females. This is in line with the results of our qPCR analysis, revealing a significant increase in *Rarres2* expression in the BAT of APOB-100 male animals. This gene encodes the adipokine chemerin, which is known to be involved in the regulation of adipogenesis and lipid accumulation [28,40]. It is reasonable to suppose that excessive lipid accumulation influences the normal function of BAT. Indeed, the level of *Ucp1* expression in the BAT of APOB-100 males was only 5% of the expression level detected in WT males. In contrast, APOB-100 females showed a two-fold *Ucp1* expression in BAT compared to WT females. As a result, in female APOB-100 animals, a dramatically (30-fold) elevated level of *Ucp1* mRNA expression was detected in BAT compared to APOB-100 males. This phenomenon may contribute to a more efficient utilization of excess triglycerides in hyperlipidemic female animals.

Obesity and related metabolic abnormalities can be prevented by regular physical activity, however, data are lacking on the exact mechanisms by which training exerts its positive effects. Exercise training influences the homeostasis of many cell types in various organs, inducing acute and chronic adaptation mechanisms. However, the exact nature and extent of the response to exercise training in various tissues, as well as the mechanisms by which different organs communicate to coordinate the appropriate response in the long run have not been clarified [41]. Therefore, our study aimed to analyze exercise-induced alterations of glucose and lipid metabolism, as well as the altered morphology and gene expression patterns of the liver and of the white and brown adipose tissues in hyperlipidemic mice.

Based on our findings, daily training efficiently reduced the extent of obesity in HFD-fed APOB-100-overexpressing mice, although it could not completely restore a normal, healthy body weight. Probably as a consequence of relatively reduced fat mass, *Tnf* and *Il1b* expression levels were significantly decreased in the subcutaneous WAT of regularly trained APOB-100 males, and also a reduced serum TNFα level was evident compared to the sedentary group. However, the leptin levels of regularly trained APOB-100 animals were unaffected, both in the adipose tissues and in the serum. Interestingly, no differences between the sedentary and trained APOB-100 groups could be detected regarding fasting glucose levels, suggesting that being overweight has a more pronounced effect on glucose metabolism than regular exercise does. On the other hand, hepatic lipid accumulation was significantly alleviated by physical training, as demonstrated by a less pronounced increase in liver weight and a remarkable relative reduction in lipid droplet number and size compared to sedentary controls.

Regular exercise training is also known to influence the thermogenic activity of adipose tissues. Indeed, our qPCR results support that the expression levels of thermoregulatory markers are increased in the subcutaneous WAT of regularly trained, healthy male animals, confirming previous observations about exercise-induced browning of WAT [6,42]. In contrast, BAT showed decreased levels of mRNA for *Ucp1* and *Cidea* in the same group of animals. Similar antagonistic effects of exercise training on the thermogenic activity of subcutaneous WAT and BAT were found by Wu and coworkers, suggesting that shifting thermogenesis from the core bodily areas to peripheral adipose tissues located closer to body surface might help to deal with further exercise-induced heat generation [6,43]. Surprisingly, we could not detect the same phenomenon in our HFD-fed transgenic mice, where *Ucp1* expression rather showed contrary, although non-significant, changes.

Mass spectrometry-based lipidomics is considered as a powerful “omics” tool which provides valuable information for a better understanding of cellular metabolism. The direct injection-based “shotgun” strategy has recently proved its suitability in analyzing adipose tissue lipidomes [44]. In our present study, a comprehensive shotgun lipidomic analysis revealed that hyperlipidemia caused multiple changes throughout the BAT lipidome, affecting both membrane and storage lipids. Importantly, hyperlipidemia essentially remodeled the membrane lipidome in a dominantly TG-rich tissue, exerting a major impact on the separation of the different treatment groups, as demonstrated by multivariate statistical analysis (PLS-DA).

Curiously, the enrichment in the amount of PG/BMP represented the largest difference among membrane lipids at the lipid class level. Under normal conditions, both PG and BMP account for 1–2% of phospholipids in most animal tissues. PG and BMP, regarded as mitochondrial and lysosomal signature lipids, respectively, are structural isomers and display versatile but fairly different functions in cells. As they possess isobaric structures, we could not distinguish them in our shotgun lipidomics analysis. Nevertheless, a recent work has mapped the tissue distribution of PG and BMP, and proved the presence of both structures in rodent fat [45]. Therefore, the detected accumulation probably affected both lipid types, and literature data indicate that both might have relevance in the context of hyperlipidemia. The increase in PG level itself might result in suboptimal mitochondrial function. In addition, the species composition of PG is determined post-synthetically by fatty acyl remodeling enzymes, such as the acyltransferase LPGAT. Lysophosphatidylglycerol acyltransferase (LPGAT) deficiency led to hepatopathy, insulin resistance, and non-alcoholic fatty liver disease in mice due to ER stress and mitochondrial dysfunction [46]. Furthermore, PG is the biosynthetic precursor of CL, the hallmark lipid of the mitochondrion. In spite of the large variation in PG level, total CL concentration was unaffected in the current model. Nevertheless, the applied long-term intervention significantly altered its species profile, although it should be noted that the relative amount of the major tetralinoleyl CL species was retained in males. Altogether, the detected changes in PG and CL might affect the functions of proteins in the inner mitochondrial membrane. As BMP is almost exclusively located in late endosomes/lysosomes, its increase might represent the role of autophagy/lipophagy in obesity [47,48]. A carnitine decrease might be associated with increased fatty acid oxidation known to occur with a high-fat diet [48].

At the lipid species level, the general increase in 18:1-containing species was in accordance with the abundance of oleic acid in the applied diet formulation. Furthermore, we detected a general loss in linoleic acid-containing species, whereas a significant increase was observed for the arachidonic acid-containing components, despite a considerable linoleic acid content and a very low arachidonic acid content of the applied HFD (Table S7). Therefore, it is suggested that linoleic acid was utilized for the synthesis of arachidonic acid followed by its esterification into membrane lipids, mainly to form the two most abundant BAT phospholipids, PC and PE. This hyperlipidemia-induced formation of arachidonic acid might serve the maintenance of membrane fluidity by counteracting the simultaneous increase in oleic acid and decrease in linoleic acid contents. On the other hand, arachidonic acid accumulation might represent a readily available pool for the formation of inflammatory signaling molecules under hyperlipidemic conditions.

The observed sex-specific lipidomic difference was dominated by the higher level of arachidonic acid-containing species in the female groups, which is in accordance with a previous report [49]. In several respects, the detected hyperlipidemic response was highly similar in male and female mice, both in direction and in extent. However, it seems that the initial, lipidome-wide sex-specific difference detected in the WT groups could affect the physicochemical properties of BAT cellular membranes, and thereby it may regulate BAT function in a sex-specific manner. For example, it might contribute to the completely different response regarding the expression of BAT *Ucp1* in hyperlipidemic mice (i.e., strong downregulation in males vs. remarkable upregulation in females). The simultaneous comparison of the lipidomes for sedentary WT, sedentary APOB-100, and regularly trained APOB-100 groups revealed that hyperlipidemia-related deterioration of BAT lipid composition was partially restored by exercise in both sexes, but this occurred in a sex-specific manner, i.e., the spectrum of affected species was largely non-overlapping in males and females.

## 4. Materials and Methods

### 4.1. Animals and Treatments

All animals were handled in accordance with the standards established by the EU Directive 2010/63/EU, and all animal experiments were approved by the regional Station for Animal Health and Food Control (Csongrád County, Hungary; project license: XVI/766/2018). Two to three mice were housed per cage, with food and water were available ad libitum. All mice were kept in the same room under controlled conditions (24 °C, 12 h light–dark cycle) throughout the experiment. The APOB-100 transgenic mouse strain overexpressing the human APOB-100 protein was previously established by our group [50] and was maintained on a C57BL/6 genetic background in a hemizygous form. In order to determine the genotype of hemizygous APOB-100 transgenic animals and wild-type littermates, DNA from tail biopsies of 10-day-old pups was purified, and the presence of the transgene was detected by PCR, using primers made for the 5′ promoter region of the human *APOB* gene [51].

As the disease model, we used APOB-100 transgenic mice fed with a high-fat diet (HFD) at 4 g/mouse/day (Special Diet Services, Witham, UK), for detailed diet composition, see Table S7), while the healthy controls were wild-type mice on a normal chow diet. They were randomly divided into sedentary and exercise groups (Figure S1A). The exercise group was trained by treadmill running, five times a week, for 45 min per occasion, at a speed of 0.9 km/h. Before the experiment, the animals were accustomed to the treadmill first and to the training afterward: starting from 0.5 km/h, the speed was increased by 0.1 km/h per week until reaching the final speed of 0.9 km/h. At the beginning and end of daily training, the speed was gradually increased/decreased by 0.1 km/h per minute. The treatments started at the age of 3 months and lasted for 7 months. Each experimental group included 12 animals. Body weight and serum glucose level (Accu-Chek, Roche, Mannheim, Germany) were measured at baseline and monthly thereafter (Figure S1B).

At the end of the experiment, all mice were terminally anesthetized by 150 µg/g sodium pentobarbital administered as an intraperitoneal injection. Simultaneously with terminal anesthesia, blood samples were collected through a cardiac puncture, and then transcardial perfusion was performed with 0.9% sodium chloride in 0.01 M PBS, pH = 7.4. After one hour of incubation at 37 °C, blood samples were centrifuged at 4 °C, 1000× *g* for 10 min, then the serum was separated and stored at −80 °C until use. Following transcardial perfusion, the liver, the brown adipose tissue, and the subcutaneous and abdominal white adipose tissues were removed. Weights of the liver and brown adipose tissue were measured, and finally, the samples were frozen in liquid nitrogen for RNA isolation or were fixed using 4% paraformaldehyde in 0.1 M PBS, pH 7.4.

### 4.2. Measurement of Serum Triglyceride

The measurement of serum triglyceride (TG) levels was performed in triplicate, and carried out using a commercially available enzymatic colorimetric assay kit (Diagnosticum Ltd., Budapest, Hungary) according to the manufacturer’s instructions. Accuracy was tested using Standard Lipid Controls (Diagnosticum Ltd., Budapest, Hungary). Serum TG concentration was determined by measuring the absorbance of the resulting purple color product at 505 nm with a microplate reader (FLUOstar OPTIMA, BMG LABTECH, Ortenberg, Germany). Values were expressed in mmol/L.

### 4.3. Measurement of Serum Leptin, Resistin, Insulin, TNF-α Concentrations

Serum fractions were collected, pooled, and stored at –80 °C before running the assay. Luminex xMAP technology was used to determine the concentrations of 4 distinct proteins (leptin, resistin, insulin, TNF-α), by performing a MILLIPLEX^®^ Mouse Adipokine Multiplex Immunoassay (MADKMAG-71K, Merck Millipore, Darmstadt, Germany) according to the instructions of the manufacturer. Briefly, all samples were thawed, centrifuged (1000× *g*, 5 min), and 10 μL volume of each sample were loaded into a 96-well plate (provided with the kit) containing mixed beads conjugated with the capture antibody. After an overnight incubation (4 °C) and thorough washing, biotinylated detection antibodies were added for 30 min. Following incubation with streptavidin–phycoerythrin (30 min) and washing, the plate was developed on a Luminex MAGPIX^®^ instrument. Luminex xPonent v4.2 software (Luminex Corporation, Austin, TX, USA) was used for data acquisition. Five-PL regression curves were generated to plot the standard curves for all analytes by Analyst 5.1 software (Merck Millipore, Darmstadt, Germany), calculating with bead median fluorescence intensity values. Results are given in pg/mL. For samples where the concentration of the given protein did not reach the detection limit, the concentration value was recorded as the lowest detection limit to make a statistical comparison with the higher concentrations. The detection ranges for the molecules were as follows: leptin 3.64–91,260 pg/mL; resistin 1.71–77,470 pg/mL; insulin 4.05–100,102 pg/mL; TNF-α 2.7–91,963 pg/mL.

### 4.4. Monitoring Gene Expression Differences in Liver and Adipose Tissues Using qPCR

RNA was isolated from the liver, as well as from the brown and white adipose tissues, with an RNA and protein purification kit (Macherey-Nagel, Düren, Germany) according to the manufacturer’s instructions. A High Capacity cDNA Reverse Transcription Kit (Thermo Fisher Scientific, Waltham, MA, USA) was used to convert mRNA samples to cDNA. Each reaction mixture contained 1 µg RNA (15 µL), 1.5 µL reverse transcriptase, 3 µL primer, 1.2 µL dNTP, 3 µL buffer, 6.3 µL RNase-free water. Parameters for the reverse transcription program were the following: incubation at 25 °C for 10 min, reverse transcription at 37 °C for 2 h, and inactivation at 85 °C for 5 min (using MJ Mini—Personal Thermal Cycler, BioRad, Hercules, CA, USA). The cDNA product was finally diluted 1:20, and was used as a template in the qPCR reaction. For the qPCR reaction, 10 µL cDNA, 1 µL (250 nM final) primer mix (forward + reverse), and 10 µL Power SYBR Green PCR Master Mix 2x (Thermo Fisher Scientific, Waltham, MA, USA) were mixed. Each reaction was performed in a total volume of 20 µL, and was run on a RotorGene 3000 instrument (Qiagen, Hilden, Germany) with the following settings: heat activation at 95 °C for 10 min; followed by 40 cycles of denaturation at 95 °C for 15 s, annealing at 60 °C for 60 s. Melting curve analysis was performed from 50–95 °C to verify the specificity of the amplification. Primer sequences used in qPCR reactions are listed in Table S8, of which the mouse *Gapdh* (glyceraldehyde 3-phosphate dehydrogenase) gene served as an internal control for normalization. Relative gene expression levels were calculated using the ΔΔCt method.

### 4.5. Adipose Tissues and Liver Pathology

Formalin-fixed tissue samples were embedded in paraffin, and 3 µm tissue sections were prepared. Sections were stained with hematoxylin–eosin to analyze the structure of the liver and adipose tissues. The number and size of the lipid droplets were analyzed using ImageJ 1.53e software (Bethesda, MD, USA).

### 4.6. Lipidomic Workflow

Lipid standards were from Avanti Polar Lipids (Alabaster, AL, USA) and Cambridge Isotope Laboratories (Tewksbury, MA, USA). Solvents for extraction and MS analyses were liquid chromatographic grade (Merck, Darmstadt, Germany) and Optima LC-MS grade (Thermo Fisher Scientific, Waltham, MA, USA). All other chemicals were of the best available grade, purchased from Sigma-Aldrich or Thermo Fisher Scientific.

A piece of BAT tissue (typically between 20–40 mg) was homogenized in water using a bullet blender homogenizer (Bullet Blender Gold, Next Advance, Inc., Averill Park, NY, USA) in the presence of zirconium oxide beads (0.5 and 1 mm), at a speed level of 10 for 3 min at 4 °C. A portion of the homogenate (corresponding to 1 mg wet weight) was immediately subjected to simple one-phase methanolic lipid extraction [52]. First, the homogenate was sonicated in 1 mL methanol containing 3 μg di20:0 PC (as an extraction standard) and 0.001% butylated hydroxytoluene (as an antioxidant) in a bath sonicator for 5 min, then shaken for 5 min and centrifuged at 10,000× *g* for 5 min. The supernatant was transferred into a new Eppendorf tube and stored at −20 °C until mass spectrometry analysis.

For mass spectrometry measurements, 5 μL of lipid extract were diluted with 145 μL infusion solvent mixture (chloroform:methanol:iso-propanol 1:2:1, by vol.) containing an internal standard mix (73 pmol PC d31-16:0/18:1, 5 pmol SM d18:1/17:0, 5 pmol DG 15:0-d7-15:0, 587 pmol TG 15:0-18:1-d7-15:0, 15 pmol carnitine, 4 pmol acetylcarnitine, and 25 pmol PE d31-16:0/18:1, 11 pmol PI d31-16:0/18:1, 20 pmol PS d31-16:0/18:1, 2 pmol PG d31-16:0/18:1, 1 pmol PA d31-16:0/18:1, 2 pmol CL t14:0, 2 pmol Cer d18:1/17:0, and 11 pmol FFA 19:0). Next, the mixture was halved, and 5% dimethylformamide (additive for the negative ion mode) or 4 mM ammonium chloride (additive for the positive ion mode) was added to the split sample halves.

Mass spectrometric analyses were performed on an Orbitrap Fusion Lumos instrument (Thermo Fisher Scientific, Bremen, Germany) equipped with a TriVersa NanoMate robotic nanoflow ion source (Advion BioSciences, Ithaca, NY, USA) using chips with spraying nozzles with a diameter of 5.5 μm. The ion source was controlled by Chipsoft v8.3.1 software. Ionization voltages were +1.3 kV and −1.9 kV in positive and negative mode, respectively, and the back pressure was set at 1 psi in both modes. The temperature of the ion transfer capillary was 260 °C. Acquisitions were performed at mass resolution R*_m/z_*_200_ = 240,000. Phosphatidylcholine (PC), lysophosphatidylcholine (LPC), sphingomyelin (SM), diacylglycerol (DG), triacylglycerol (TG), carnitine (Carn), and acetylcarnitine (AcCar) were detected and quantified using the positive ion mode, whereas phosphatidylethanolamine (diacyl, PE and alkenyl-acyl, PE-P), phosphatidylinositol (PI), phosphatidylserine (PS), phosphatidic acid (PA), phosphatidylglycerol (PG), cardiolipin (CL), and the lyso derivatives LPE, LPI, LPS, LPG, LPA, and LCL, as well as ceramide (Cer) and free fatty acid (FFA), were detected and quantified using the negative ion mode. Lipid species were identified by LipidXplorer 1.2.8.1 software [53]. Identification was executed by matching the *m*/*z* values of their monoisotopic peaks to the corresponding elemental composition constraints. Mass tolerance was set to 2 ppm. To resolve fatty acyl composition in glycero(phospho)lipids, data-dependent tandem MS2 or MS3 fragmentation experiments were performed based on mass lists from survey scans. The coexistence of PG and BMP species was confirmed by MS/MS fragmentation experiments in positive ion mode [54]. Data files generated by LipidXplorer queries were further processed by in-house Excel macros.

To annotate lipid classes and species, we applied the classification systems for lipids [55]. Sum formulas for glycero(phospho)lipids are defined as the lipid class abbreviation followed by the total number of carbons and total number of double bonds for all chains, e.g., PC(34:1) or TG(54:3).

### 4.7. Statistical Analysis

Two-way analysis of variance (ANOVA), followed by the Holm–Šídák post hoc test, was performed using SigmaPlot12 software (Systat Software Inc., San Jose, CA, USA). The level of statistical significance was set at *p* < 0.05. Data were expressed as mean ± SEM. Lipidomic data are presented as mol% of membrane lipids, where membrane lipids include glycerophospholipids and sphingolipids but exclude DG, TG, FFA, and carnitines, and are expressed as mean ± SEM. ANOVA, followed by Student’s *t*-tests, was performed for pairwise multiple comparisons. False discovery rate (*q* value) was determined according to the Storey–Tibshirani method [56]; significance was accepted for *p* < 0.045 corresponding to a false discovery rate *q* < 0.05. Partial least squares and sparse partial least squares discriminant analyses (PLS-DA and sPLS-DA, respectively) on lipidomic datasets were performed using MetaboAnalyst [57].

## 5. Conclusions

Our results confirm that HFD-treated APOB-100 transgenic male mice represent a suitable model to study not only cardiac dysfunction, but also several symptoms and consequences of metabolic syndrome, such as abdominal obesity, dyslipidemia, increased fasting glucose, or hepatic steatosis. As another important finding, we provide evidence that despite the same diet, there are remarkable differences between male and female animals, regarding, for example, fasting glucose, serum triglyceride levels, gene expression, and lipidomic patterns. This may be explained by the protective effects of estrogen and the sex-dependent differences in the regulation of energy metabolism. Our findings indicate that daily exercise training efficiently reduced the rate of increase in body weight and serum triglyceride level, as well as the expression of pro-inflammatory factors in adipose tissues, and also reduced hepatic lipid accumulation in our model. However, it seems that exercise alone is insufficient to eliminate all the alterations induced by an HFD, as fasting glucose level did not decrease in response to training.

## Data Availability

All data generated or analyzed during this study are included in this published article and its supplementary information files.

## Supplementary Materials

The following are available online at https://www.mdpi.com/article/10.3390/ijms22084198/s1.
