# Supplementary material for: Male and Female Animals Respond Differently to High-Fat Diet and Regular Exercise Training in a Mouse Model of Hyperlipidemia
Source: Int J Mol Sci. 2021 Apr 18;22(8):4198. doi: 10.3390/ijms22084198 (PMC8073713; doi:10.3390/ijms22084198)
Supplement: Supplementary file 1 [file ijms-22-04198-s001.zip › ijms-1176662-supplementary/Supplementary figures revised.pdf]

Supplementary Materials

Male and female animals respond differently to high-fat diet and regular exercise training in a mouse model of hyperlipidemia

Tóth M.E.[1*#] Dukay B.[1,2#], Péter M.[1], Balogh G.[1], Szűcs G.[3], Zvara Á.[4], Szebeni G.J.[4], Hajdu P.[1], Sárközy M.[3], Puskás L.G.[4], Török Z.[1], Csont T.[3], Vígh L.[1] and Sántha M.[1]

[1] Institute of Biochemistry, ELKH Biological Research Centre, Szeged, Hungary,
[2] Doctoral School in Biology, University of Szeged, Szeged, Hungary,
[3] MEDICS Research Group, Department of Biochemistry, Interdisciplinary Center of Excellence, University of Szeged, Szeged, Hungary,
[4] Laboratory of Functional Genomics, ELKH Biological Research Centre, Szeged, Hungary
[#] These authors contributed to the work equally.
* Corresponding author

A

Genotype/diet	Exercise	Sex	No. of animals
Wild type/ normal diet	sedentary	male	12
		female	12
	exercise	male	12
		female	12
APOB-100/ High-fat diet	sedentary	male	12
		female	12
	exercise	male	12
		female	12

B

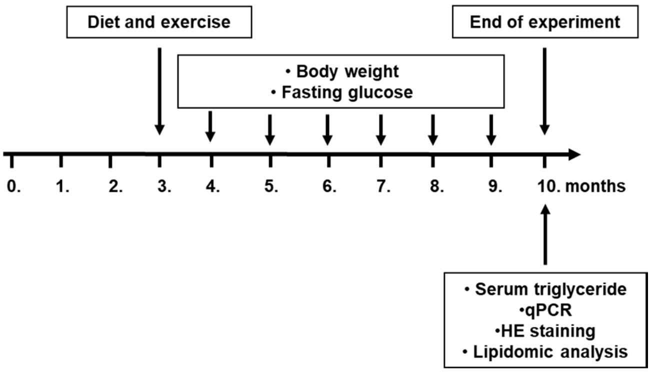

Figure S1. (a) Experimental groups and **(b)** schedule of the experiment.

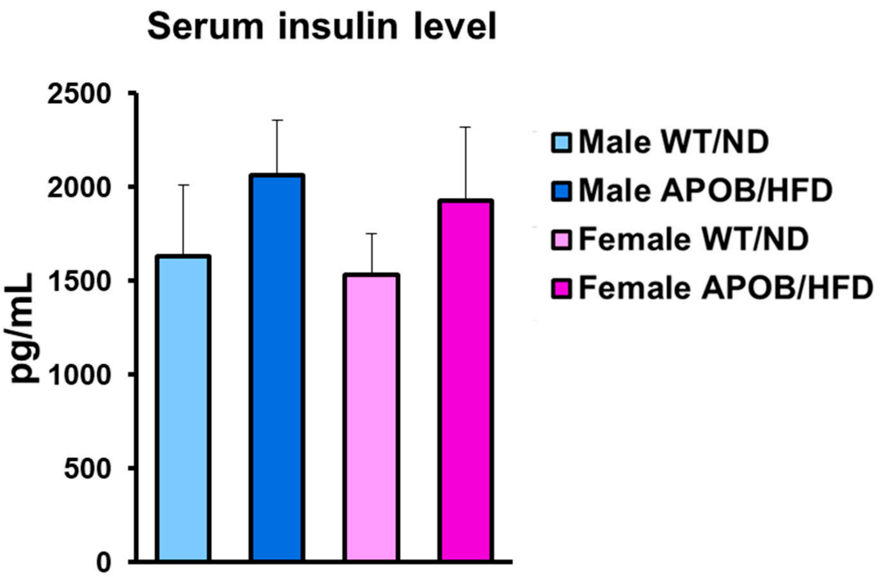

Figure S2. Serum insulin concentration quantified using a MILLIPLEX® Mouse Adipokine Multiplex Immunoassay (n=9-10 animals/group).

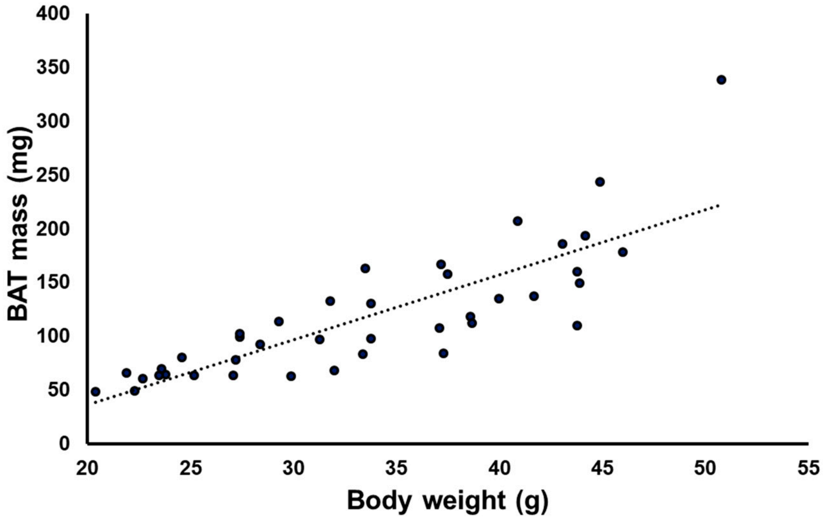

Figure S3. Correlation between body weight and BAT mass in sedentary groups. r=0.816, *P*=1.5E-11.

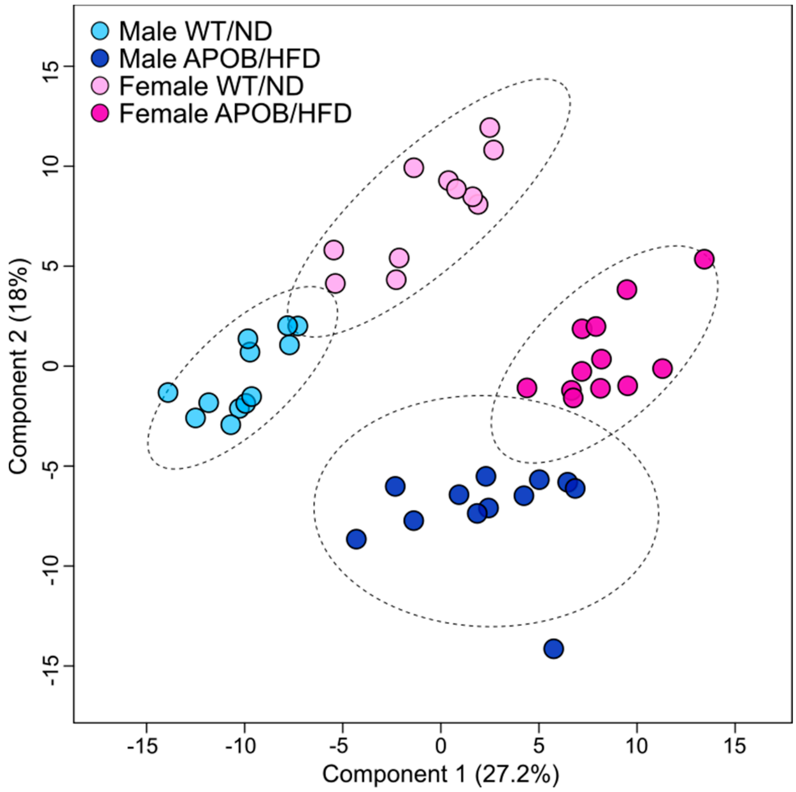

Figure S4. PLS-DA scores plot of lipidomic datasets based on the membrane lipids only.

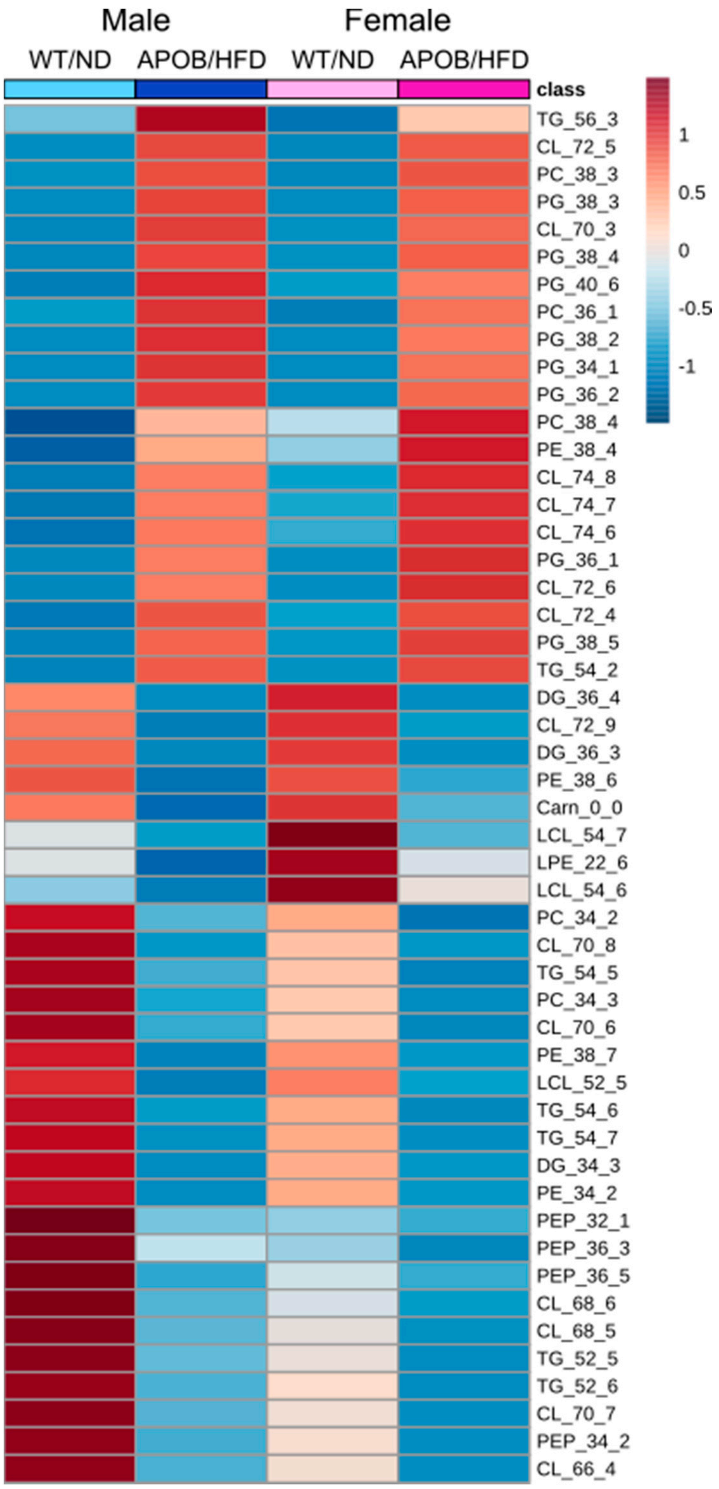

Figure S5. Heatmap representation of hierarchical cluster analysis of lipidomic datasets for sedentary WT/ND and APOB/HFD groups. Top 50 most significant species were selected based on ANOVA; distance measure, Euclidean; clustering algorithm, Ward; heat color code represents normalized values (z-scores).

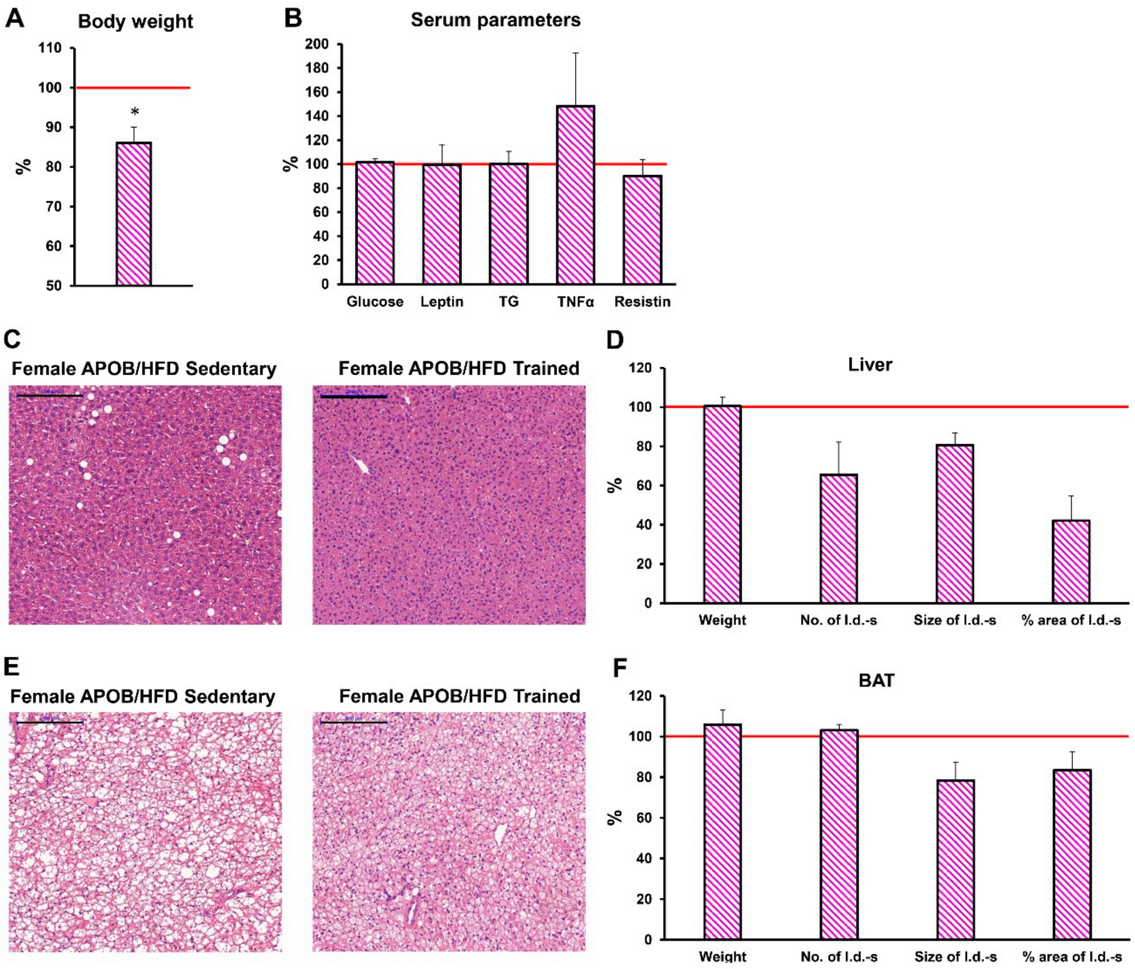

Figure S6. Effects of exercise training in HDF-fed female APOB-100 transgenic mice. Magenta striped columns represent the differences in the given value in regularly trained APOB100/HFD female mice, expressed as the percentage of the corresponding value for sedentary APOB100/HFD females. Scale bar: 200 µm. n = 12 mice per group for body, liver, and BAT weight and serum glucose measurements, n = 10 mice per group for serum leptin, TNFα, and resistin concentration measurements, n = 6 mice per group for the quantification of lipid accumulation. Values are presented as mean ± SEM; * denotes $P<0.05$; WT/ND, wild type mice on normal diet; APOBHFD, APOB-100 transgenic mice on high-fat diet; BAT, brown adipose tissue; l.d., lipid droplet.
